# The implementation of neurologic teleconsultations in rural primary care in Northeastern Germany (NeTKoH): an evaluation guided by the RE-AIM framework

**DOI:** 10.1186/s12913-026-16062-0

**Published:** 2026-11-10

**Authors:** Kerstin Wainwright, Hans-Aloys Wischmann, Paula J. Filser, Ricarda S. Schulz, Simone Kiel, Jean-François Chenot, Agnes Flöel, Anselm Angermaier, Felix von Podewils, Batya O. Elul, Tobias Kurth

**Affiliations:** 1 Institute of Public Health, Charité – Universitätsmedizin Berlin, Berlin, Germany; 2 Department of Health Care Management, Technische Universität Berlin, Berlin, Germany; 3 Institute for Community Medicine, Department of General Practice, University Medical Center Greifswald, Greifswald, Germany; 4 Department of Neurology, University Medical Center Greifswald, Greifswald, Germany; 5 Fachkrankenhaus für Psychiatrie, Psychosomatik und Psychotherapie, Evangelisches Krankenhaus Bethanien gGmbH, Greifswald, Germany; 6 Department of Epidemiology, Mailman School of Public Health, Columbia University, New York, USA

**Keywords:** Implementation, RE-AIM, Teleneurology, Teleconsultation, Primary care, General practitioner, Population health, Care management

## Abstract

**Background:**

Neurologic diseases represent a major public health burden both globally and nationally, straining the health care systems especially in rural areas with a scarcity of neurologists, and endangering patients’ proximal access to care. The NeTKoH project (Neurologic Teleconsultation with General Practitioners to strengthen specialist care in Western Pomerania) addressed this by providing teleconsultations with neurologic experts from a university hospital for patients presenting with neurologic symptoms in primary care practices in rural areas of Western Pomerania in Northeast Germany.

**Objective:**

A comprehensive evaluation of such teleneurologic interventions under real-world conditions, focusing on their generalizability (both internal and external validity), is essential to advise decision-makers. We applied the Reach-Effectiveness-Adoption-Implementation-Maintenance (RE-AIM) framework to systematically assess NeTKoH’s implementation and to inform its translation from research to practice.

**Methods:**

Each RE-AIM dimension was operationalized: ​*Reach* comprised the proportions of patients enrolled out of those approached by their physicians and of patients in the intervention who received a teleconsultation; *Effectiveness* measured the proportions of patients triaged to general practitioner care, of patients with improved QoL at follow-up, of same-day teleconsultations, and of positive patient responses regarding satisfaction; *Adoption* assessed the proportions of participating and of continuously participating physicians; *Implementation* focused on installed devices, preparatory visits with device training, device use by physicians, and teleconsultations performed; *Maintenance* measured patients’ and physicians’ self-reported intention to continue using teleconsultations.

**Results:**

NeTKoH demonstrated high scores for *Reach* (98.8%), *Adoption* (83.0%), *Implementation* (99.2%), and *Maintenance* (85.0%), while *Effectiveness* was “generally positive” (66.0%). The representativeness of patient and physician populations in the study was limited, but both were broadly comparable to the regional target populations.

**Discussion/Conclusion:**

Our findings suggest that teleconsultations for patients with neurologic conditions in primary care settings are feasible, acceptable, and improve access to specialist care. The RE-AIM framework provided relevant insights that can guide a dissemination trial for a potential scale-up.

**Trial registration:**

German Clinical Trials Register (DRKS00024492), registered on September 28, 2021.

**Supplementary Information:**

The online version contains supplementary material available at https://doi.org/10.1186/s12913-026-16062-0.

## Introduction

Telemedicine is increasingly recognized for its cross-disciplinary and cross-sectoral potential, especially in addressing care disparities and enhancing access to healthcare services in underserved regions [1]. It is defined as “the use of electronic and telecommunication technologies to provide healthcare at a distance” [2]. This approach has shown promise in improving care quality in rural areas where access to specialized medical expertise is limited [1, 3, 4]. It gained particular momentum during the COVID-19 pandemic, which drove a marked and sustained increase in the use of video consultations in general practice internationally [5]. Over the past two decades, telemedicine has been applied extensively within neurology, referred to as teleneurology, encompassing both clinical neurosciences and acute neurologic care settings [3, 6]. In primary care, dedicated teleneurology programs have also been implemented, utilizing real-time video consultations and remote diagnostic support. These initiatives have demonstrated improvements in care coordination and quality while also achieving high satisfaction rates among patients and practitioners and saving costs [3, 7–9]. Reflecting this, the European regional network of the World Health Organization of Family Doctors (WONCA) advocated for the wider integration of telemedicine into primary care in its Statement on Telemedicine, presented at the WHO Europe 70th Regional Committee Meeting in September 2020 [10].

From a health policy perspective, this is particularly important in rural regions, such as in the state of Mecklenburg-Western Pomerania in Northeast Germany, which exhibits the lowest population density and the third-oldest population demographic in Germany [11–13]. Here, outpatient care is mainly provided by general practitioners (GPs), with a markedly low density of specialists, including neurologists, per person at risk. The growing demand for neurologic expertise, driven by demographic change, places increased strain on the healthcare system, which is facing substantial challenges in ensuring that timely specialist care is available nearby, especially in states with low population density.

Therefore, the NeTKoH project aimed to integrate neurologic teleconsultations into routine primary care workflows through a network approach between the local university hospital and GPs, to improve access of underserved communities in Western Pomerania to neurologic specialists at the university level, with the potential to serve as a model for scale-up to all of Germany.

A thorough evaluation of innovative interventions under real-world conditions requires an assessment of both efficacy and generalizability, i.e., both internal and external validity, and of their potential for integration into everyday practice [14–16]. The Reach-Effectiveness-Adoption-Implementation-Maintenance (RE-AIM) framework [14] offers a systematic approach for such an evaluation and can assess the potential for translation from research into practice. We chose the RE-AIM approach as it is one of the most frequently applied implementation frameworks and has been used widely for diverse areas and settings [17]. Therefore, the objective of this study is to evaluate the implementation of neurologic teleconsultations in GP practices in Northeast Germany, using the RE-AIM approach to disseminate experiences and to provide insights for a potential scale-up.

## Methods: description

### Design

NeTKoH was designed as a stepped wedge cluster-randomized trial with continuous recruitment in routine primary care over a period of 36 months and 18 days with 11 steps, where neither physicians nor patients could be blinded to the intervention [18]. The teleneurologic intervention was introduced stepwise across 33 GP practices, where clusters of 3 to 4 practices per step transitioned from control (standard care) to intervention (teleconsultation) in parallel. To power the study appropriately, an enrollment of 3 patients on average per step per GP was required [18]. Patients received the standard of care including standard referrals and treatment (control phase) or the teleconsultation (intervention phase), according to the phase of the respective GP practice at the time of the index visit/inclusion.

Given the scope and complexity of the NeTKoH evaluation, the trial and its endpoints, implementation, health economic outcomes, and detailed patient satisfaction are reported separately. The trial and its primary and secondary endpoint results have been published previously [19, 20], as well as the findings of the health economic study [21, 22], while a detailed patient satisfaction analysis will be reported separately. The present study focuses on the implementation of NeTKoH during the intervention phase, including patients enrolled from April 2022 through October 2024. Overall, our approach to NeTKoH can be classified as a hybrid design. We used the RE-AIM framework [14] to guide the evaluation and followed the Standards for Reporting Implementation Studies (StaRi) [23] and the expanded RE-AIM CONSORT figure [24].

### Context, sites, and eligibility

The NeTKoH study was conducted in an outpatient primary care setting in rural Western Pomerania in the state of Mecklenburg-Western Pomerania in Northeast Germany.

Recruitment of the GPs started in 2020, coinciding with the COVID-19 pandemic, and patient enrollment was planned for 2021. As a first step to recruit GPs, the local Association of Statutory Health Insurance Physicians and the University Medical Center Greifswald (UMG), the regional university hospital, sent an introductory letter about the project to GPs in the target region. GP recruitment proved to be challenging because of the pandemic and a shift in the national vaccination strategy to GPs practices. Ten months before the trial’s launch, informational sessions about the intervention were conducted. GPs who expressed interest via a Letter of Intent were subsequently visited by members of the NeTKoH project team. During these visits, the study design was presented in detail, and supplementary materials, including a handout, were distributed. Following this, cooperation agreements were signed. In October 2021, patient recruitment for the trial started.

To encourage participation, each GP received 200€ for the initial visit before the start of the study and 200€ for the preparatory visit, as they needed to close their practice for about 2 h during the installation of the telemedicine device. In addition, GPs received an incentive of 100€ per enrolled patient to promote recruitment, but the study did not provide any further financial compensation, e.g., for staff time in the GP practices to deliver the intervention. However, an additional position for a dispatcher, a coordinator, and a neurologist to establish and perform the video-consultations was funded at the participating university hospital. Equipment for the video-conferencing system was provided and maintained at no cost to the sites by the NeTKoH consortium partner MEYTEC GmbH (Werneuchen OT Seefeld, Germany).

Eligible study sites had to be (1) located in the region of Western Pomerania, (2) managed by GPs or specialists in internal medicine, who (3) saw patients with statutory health insurance, and (4) met the technical and space requirement for teleconsultations. Eligible patients had to (1) be at least 18 years of age, (2) present at a participating GP practice with neurologic symptoms (that might warrant a referral to a neurology specialist), (3) have statutory health insurance, and (4) be able to give informed consent [25]. Given the wide range of neurologic symptoms and the impracticality of using standardized checklists in routine primary care, inclusion was based on the GP’s assessment of a deficit that might warrant referral to a neurologist and the question: “ Would you desire a neurologic teleconsultation for this patient?”.

### Intervention and implementation strategies

The NeTKoH project aimed to establish a new form of care within the German healthcare system for patients with neurologic symptoms by facilitating timely teleconsultations between GPs, their patients, and neurology specialists.

Using the NeTKoH teleconsultation system, the GP and patient were connected in real-time with a teleneurologist, enabling audiovisual remote interaction. Teleconsultations were intended to be conducted during or as soon as possible following the GP visit, with one teleconsultation per patient. The NeTKoH system was a hardware-based video conferencing solution, featuring integrated microphones, speakers, and a remote-controlled camera. It operated independently from the practice’s internet connection via the mobile network. The system enabled encrypted video communication and data transmission and was linked to the teleneurology database (Tenedoc), which supports the HL7 communication standard. During regular GP consultation hours (Monday to Friday, 8 a.m. to 5 p.m.), the university hospital’s Department of Neurology offered real-time teleneurology appointments and prompt specialist-level consultations for participating GPs. The intervention was designed to integrate smoothly into daily practice routines, minimizing disruptions.

When a patient presented with symptoms suggestive of a neurologic disease that could require a consultation with a neurology specialist, the GP informed them about the study. Upon providing informed consent, in the intervention, the patient was asked to wait while a medical assistant activated the telemedicine system, ideally in a separate room. This notified a staff member (“dispatcher”) at the Neurology Department, who proposed an appointment time. The practice confirmed the appointment and informed the patient. The teleconsultations, intended to last no more than 15 min, covered topics such as prior diagnoses, symptom presentation, prior diagnostics, treatments, referrals, medications, and included a focused clinical examination with the aim of an appropriate triage. After the consultation, the teleneurologist documented the findings and recommendations electronically, ​and a printout was generated via a printer directly connected to the teleconsultation system. The implementation strategy had two key elements targeting the providers (type A [26]): the introduction and information sessions at the beginning of the project, and hands-on preparatory visits with the individual GPs a few weeks before their switch to the intervention. These visits included guidance on transitioning from the control to the intervention phase and a re-iteration of the eligibility criteria for NeTKoH patients. Medical Assistants (Medizinische Fachangestellte) of the GPs were provided with a summary of the informed consent form and an illustrative list of relevant neurologic symptoms. During these preparatory visits, approximately 4 to 6 weeks prior to the start of the intervention, the MEYDOC XS telemedicine system, a certified Class I medical device, was installed, and GPs and their staff received a technical briefing on the video-conferencing system’s practical use. The GP, their medical assistant, a neurologist, a MEYTEC representative, and a NeTKoH team member from the UMG participated.

### Outcomes: RE-AIM dimensions

We used the RE-AIM approach to assess the public health impact of NeTKoH using 5 dimensions to assess NeTKoH’s implementation in a real-world setting. RE-AIM focuses on the context, such as setting, participants, implementers, and the frequency and intensity of intervention activities [17].

#### Reach metrics and data

*Reach* is defined as “the absolute number, proportion, and representativeness of individuals who are willing to participate in the intervention relative to the broader eligible population (beneficiaries)” [27], i.e., a patient-level measure. GPs were not blinded when they screened and enrolled eligible patients based on the inclusion criteria. Thus, *Reach* was calculated as (1) the proportion of patients willing to participate in the intervention, based on the GP’s paper screening log after they approached a suitable patient and (2) the proportion of intervention patients who actually received a teleconsultation, per the Tenedoc database. Also, participant characteristics in the intervention group were compared between those who were and were not lost to follow-up (LTFU). Their representativeness was evaluated using official population statistics.

#### Effectiveness metrics and data

We assessed *Effectiveness*, which is defined as “the impact of the intervention on the intended health outcomes for a given sample or population” [27], using the primary outcome of the NeTKoH stepped wedge RCT as well as its patient-oriented secondary endpoints, including QoL and just-in-time care endpoints, as well as satisfaction with the intervention. We operationalized *Effectiveness* as (1) the proportion of intervention patients triaged to remain at their GP, i.e., their neurologic problem could be managed at the primary care level (neurologic question resolved), (2) the proportion of patients in the intervention with higher QoL index at follow-up, (3) the proportion of patients in the intervention with same-day teleconsultation, (4) the proportion of patients with 3 positive answers regarding satisfaction, acceptance, and recommendation of the intervention. Patients could instead be referred to an outpatient neurologist, another specialist, or a hospital (secondary endpoint). Referral outcomes were assessed immediately following the teleconsultations and again at the three-month follow-up and are reported in detail in the trial and endpoint publication [19]. For context, the group comparison of the primary endpoint from the pragmatic trial is also reported in the Results. Quality of life (QoL) was included as a patient-oriented secondary endpoint of the pragmatic trial and was also relevant to the health economic evaluation required by the funder. For the *Effectiveness* dimension, we used the number of patients in the intervention arm with a teleconsultation as the denominator. Data on patients remaining with their GP and on same-day teleconsultations came from the UMG Tenedoc database. Patient satisfaction and acceptance of the intervention were assessed using selected, relevant items from a self-developed, piloted paper questionnaire derived from elements of existing questionnaires that were tailored to the context [28–30], which was completed after the teleconsultation (see Supplementary Materials Table S1). Quality of life was evaluated via the German EQ-5D-5L as part of that same questionnaire [31], on paper immediately following the intervention and by phone at follow-up.

#### Adoption metrics and data

*Adoption* is defined as “the absolute number, proportion, and representativeness of settings and intervention delivery agents” [27], with a focus on understanding why an intervention was or was not adopted [17]. *Adoption* was measured using (1) the proportion of participating GPs among those who expressed interest during the recruitment and (2) the proportion of GPs who participated until the end of the project, as recorded in a UMG log. GP characteristics were assessed using a GP entry questionnaire, and their representativeness was evaluated against public health practitioner statistics. We also compared GPs who continued the intervention with those who dropped out, adding reasons for discontinuation when given.

#### Implementation metrics and data

*Implementation* refers to how closely the intervention delivery in the real world matches the original design – this includes fidelity to its core components, consistency in delivery, and considerations of time and cost [27]. *Implementation* was evaluated by (1) the proportion of preparatory visits with training on the device carried out as planned, according to the UMG log, (2) the proportion of GPs who had the video conferencing device installed as documented in the UMG Log, (3) the proportion of GPs who used the teleconsultation device at least once, assessed via the UMG Tenedoc database, and (4) the proportion of requested teleconsultations that were conducted, assessed via the UMG Tenedoc database. Furthermore, technical problems and their consequences, as well as just-in-time endpoints, were analyzed using the Tenedoc database. Finally, intervention costs were calculated.

#### Maintenance metrics and data

*Maintenance* addresses the extent to which the intervention becomes institutionalized at the setting level or sustained at the individual level [17]. To measure *Maintenance*, we used the reported intention for future use of teleconsultations by patients and GPs as an indicator, as captured in the questionnaires (see Supplementary Materials Table S1). Further details of the operationalization of the RE-AIM dimensions, the chosen numerators and denominators, and the respective data sources are shown in Appendix Table A1.

### Statistical analyses

RE-AIM dimensions and process parameters were analyzed descriptively using quantitative methods, including patient demographics at enrollment and GP demographics at project start. Categorical variables were reported as counts and percentages; continuous variables as mean with standard deviation (SD) and median with quartiles or with min/max values for age. Analyses were stratified by groups (patients, physicians). Physicians that discontinued their participation and patients who were LTFU were also described.

Based on an assumption of Missing Completely at Random (MCAR), patients with a missing primary endpoint were excluded from all outcome analyses but included in all process and implementation analyses. For data privacy reasons^1^, less than 5 persons in sex, age, or sex-age subgroups consisting of one person only were excluded. An available case strategy was used for the descriptive analyses: Unlike a complete-case approach, this strategy excludes patients only from analyses for which specific data are missing, rather than removing them from all analyses. Patients for whom no data was recorded during the 3-month telephone follow-up were formally classified as LTFU, but they were still included in all statistical analyses for which the required data were available.

## Results

**Fig. 1:**
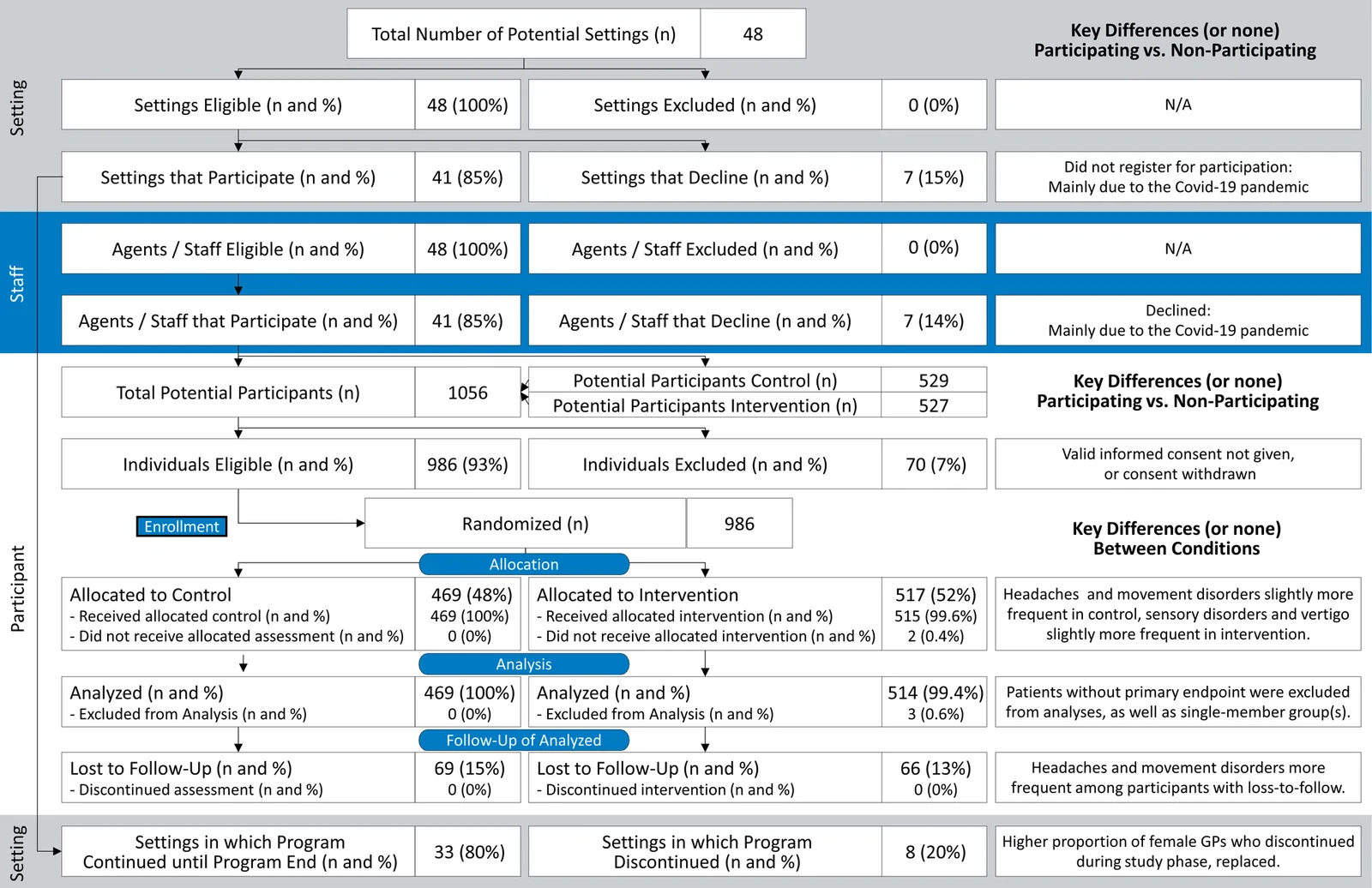
Expanded CONSORT figure RE-AIM

Over the 36-month period from Step 1 to the end of Step 11 of the NeTKoH project, a total of 1,056 patients were approached by their GPs regarding potential participation in the project and screened for inclusion. Of these patients, 527 were approached during the intervention phase. In total, 986 (93.4%) were enrolled, while 70 (6.6%) were not eligible or did not provide their consent. Of these 986 patients, 517 (52.4%) were enrolled during the intervention and 469 (47.7%) during the control phase. This slight imbalance was expected due to the detailed stepped wedge design. In total, 66 (12.8%) intervention patients and 69 (14.7%) control patients were LTFU (see Fig. 1). Reasons for non-participation could not be collected due to data protection considerations, but reasons for loss to follow-up were captured, where available.

### Reach

For *Reach* we considered (1) the proportion of patients who were willing to participate and enrolled in the study out of those approached by the GP and the (2) proportion of patients that actually received the intervention out of those who were willing to participate and enrolled. In the case of NeTKoH, a complete enumeration approach was applied to minimize identification and selection bias, so that all potentially eligible patients were indeed approached. Of the 527 patients approached during the intervention phase (unbeknown to the patients at that time), 517 (98.1%) were willing to participate and were enrolled after informed consent. Of these, 515 (99.6%) received a teleconsultation, while 2 patients did not, due to technical problems with the device. In accordance with data protection regulations, information on one person could not be reported, so that patient data could only be analyzed for 514 patients who received the intervention. The average age of the 514 patients who received a teleconsultation was 53.2 years, with an age range from 18 to 89 years, 60.1% were female. The largest proportion presented with a headache, followed by sensory disorders, vertigo, other pains, other symptoms, and motor deficits (see Appendix Table A2). Given the study design, with enrollment and teleconsultation on the same day or within very few days, and surveys immediately after the intervention, revocation of informed consent was the only source of patient dropouts, affecting less than 5 cases. Follow-up data, collected three months after the index visit, was unavailable for 66 patients (12.8%).

Demographic characteristics of patients who were and were not LTFU are presented in Table 1, and clinical symptoms for patients who were and were not LTFU are listed in Appendix Table A2.

**Table 1:** Reach – demographics of intervention patients who were and were not LTFU

Patients in the Intervention	Followed Up (*N* = 448)	Lost to Follow-Up (*N* = 66)	Total (*N* = 514)
**Sex**			
Female	270 (60.3%)	39 (59.1%)	309 (60.1%)
Male	178 (39.7%)	27 (40.9%)	205 (39.9%)
**Age [years]**			
Mean (SD)	54.4 (19.3)	45.3 (20.3)	53.2 (19.6)
Median [Min; Max]	57.0 [18; 89]	41.0 [19; 85]	56.0 [18; 89]
**Age group**			
18–29 years	58 (12.9%)	22 (33.3%)	80 (15.6%)
30–49 years	117 (26.1%)	16 (24.2%)	133 (25.9%)
50–69 years	155 (34.6%)	17 (25.8%)	172 (33.5%)
70–79 years	71 (15.8%)	6 (9.1%)	77 (15.0%)
≥ 80 years	47 (10.5%)	5 (7.6%)	52 (10.1%)

With respect to the frequency of neurologic symptoms, intervention patients who were LTFU showed a higher prevalence of headaches (36.4% vs. 32.6%), motor deficits (12.1% vs. 6.5%), and vision impairments (9.1% vs. 7.8%). In contrast, sensory disorders (27.7% vs. 22.7%) and vertigo (26.3% vs. 21.2%) were more common among patients who were not LTFU (see Appendix Table A2). For data protection reasons, results were not reported for less than five cases.

#### Representativeness of the NeTKoH intervention population

In order to maximize the potential transferability of the NeTKoH results to the federal state of Mecklenburg-Western Pomerania or to Germany (generalizability), the characteristics of the study population should align closely with contemporaneous values of the target population. The mean age of NeTKoH intervention participants was 53.2 years (SD 19.6) (see Table 1), which is somewhat higher than the general population in Mecklenburg-Western Pomerania (47.4 years) [13] and Germany overall (44.7 years) [32], but notably lower than the average age of neurologic inpatients discharged from hospitals in Mecklenburg-Western Pomerania in 2023 (approximately 65 years) [33]. The proportion of female patients both in the study overall (61.5%) and in the intervention (60.1%) exceeded that of the general population in Mecklenburg-Western Pomerania (50.9%) [13] and in Germany (50.7%) [32]. This difference is only partially attributable to the higher average age of the study population, as the proportion of women in Mecklenburg-Western Pomerania surpasses 60% only among individuals aged 82 and older [13]. Clinically, headache was the most frequent condition among NeTKoH patients in either phase, consistent with national patterns where tension-type headache and migraine are the predominant neurologic complaints [34, 35].

### Effectiveness

For *Effectiveness*, the primary outcome and the patient-oriented outcomes of QoL, just-in-time care, and acceptance were considered. Due to details of the study design, the number of patients enrolled differed slightly between control and intervention, so that the number of patients in the intervention was used as the denominator. Out of 516 intervention patients, outcomes for 514 patients were reported and analyzed, as patients without a primary endpoint and single-member sub-groups were excluded from the analyses.

Of these 514 intervention patients, the neurologic question could be resolved for 197 (38.3%), i.e., the patients were triaged to remain with their GP, followed by a similar fraction that was referred to an outpatient neurologist after the teleconsultation (36.4%), while 11.9% were referred to other specialists and a higher than anticipated proportion of 12,8% was advised to seek hospital care (see Appendix Table A3). For context, in the control phase, 50.7% of patients were triaged to remain under GP care, 41.4% were referred to an outpatient neurologist, 4.7% to another specialist, and 2.6% to a hospital [19]. For 238 patients (46.3%) in the intervention phase, the QoL index showed improvement between enrollment and follow-up, although the trial analysis showed no significant difference in QoL change between the intervention and control phase [19]. In addition, 507 (98.6%) received a teleconsultation with a neurologic specialist on the same day [19]. Regarding satisfaction with, acceptance, and recommendation of teleconsultations, 416 (80.9%) responded with positive answers, i.e., “strongly agree” or “agree” to all three questions (see also details in Appendix Table A4). Results from the expanded patient satisfaction survey will be reported separately. The corresponding questions and response options are provided in Supplementary Materials Table S1.

### Adoption

Following the three information events regarding the NeTKoH project, 40 GPs in Western Pomerania expressed interest in participating as potential settings from January 01, 2021, onward, primarily through letters of intent. During the course of the project, 8 more GPs expressed their interest in stepping in for GPs who were leaving, bringing the total number of eligible GPs to 48. However, due to the impact of the COVID-19 pandemic and the associated vaccination strategy shift to GPs, 7 of the original 40 practices did not register for the start of the NeTKoH trial phase by October 1, 2021, and another 8 withdrew at a later point in time. A total of 41 out of 48 (85.4%) GPs with interest in participating in the study enrolled at least one patient, and 33 out of 41 (80.5%) actively participated until the end of the study (settings in which program continued), defined as having enrolled at least one patient and remained in the study until its end.

GPs who participated until the end were predominantly male (63.6%), with ages ranging from 34 to 65 years, averaging around 50 years (mean 48.2, SD 9.1). Slightly more were in the < 45 age group (42.4%) than the 45–54 age group (36.4%). The average practice experience was approximately 20 years (mean 19.5, SD 9.0), of which about 10 years were in Mecklenburg-Western Pomerania (mean 9.9, SD 7.7). Approximately two-thirds of the participating GPs (69.7%) reported having no experience with telemedicine. More than half (63.6%) worked in a single practice, and half (54.5%) treated 1,000 to 1,500 cases per quarter (see Appendix Table A5).

Of the 8 GPs that left the project, 6 were female (75%). With respect to age groups, telemedicine experience, or type of practice, there were no relevant differences between GPs who continued and those that left the study. Stated reasons to discontinue participation were predominantly personal or organizational, including time constraints, staff shortages, or a lack of integration into practice routines. Some GPs left the practice or stopped practicing, and only very few gave no reasons. For data protection reasons, further details could not be reported.

#### Representativeness of the NeTKoH GPs

At the start of the project, 11 of the 33 NeTKoH GPs were female (33.3%), and after replacing the GPs that left, the proportion remained at 36.4% until the end of the project. In Mecklenburg-Western Pomerania, at the time of our study in 2023, there were around 1,200 GPs, i.e., general practitioners and specialists in internal medicine working as GPs [36], with the proportion of female GPs at 58.7% [37], much higher than in NeTKoH. At the same time, almost exactly half of GPs in Germany were female (50.5%) [38], less than in Mecklenburg-Western Pomerania, but still more than in NeTKoH. At 48.2 years, the average age of NeTKoH GPs was below the average age of GPs in Mecklenburg-Western Pomerania at 52.9 years [37] and in Germany at 55.2 years [38]. In 2023, an average of approximately 900 treatment cases per quarter was recorded nationwide in the primary care sector [39]. In Mecklenburg-Western Pomerania, the number was regularly slightly above the nationwide average [40], and the majority (54.5%) of NeTKoH GPs reported the corresponding category of 1,000 to 1,500 cases per quarter.

At the organizational level, most NeTKoH GPs (63.6%) worked in individual practices, which is similar to the physician practices in Mecklenburg-Western Pomerania in 2023 at 64.8% [41]. Thus, the NeTKoH GPs were similar in terms of practice type and average number of cases per quarter but were younger and more frequently male than in Mecklenburg-Western Pomerania or in Germany.

### Implementation

#### Preparatory visits and MEYTEC devices

Overall, devices were installed in 34 practices, some of which served multiple GPs, and some of which were installed for GPs that later left. Of these 34 practices, all (100.0%) received preparatory visits which included training on the MEYTEC teleconsultation device. Teleconsultations were actually carried out by 32 (97.0%) out of 33 GPs that participated at any given point in time. Of the 516 desired teleconsultations, a total of 514 (99.6%) were performed.

#### Technical problems

Technical problems occurred in 131 (25.5%) of the 514 teleconsultations performed (see Table 2). Detailed information on 22 initial cases was unavailable, as the documentation in Tenedoc database was only upgraded after the start of the intervention. Of the 109 documented cases with technical issues, the issues affected the GP site in 65 cases (59.6%), and the teleneurologist site in 67 (61.5%), and thus some affecting both. Audio problems were more frequent (85 cases, 78.0%) than video problems (60 cases, 55.0%), and only one teleconsultation was terminated because of technical problems. Otherwise, the problems mainly led to a longer teleconsultation duration in 105 cases (80.2%), and no recorded impact in 25 cases (19.1%).

**Table 2:** Implementation – technical problems during teleconsultations

Teleconsultations initiated (N = 514)	
**Technical problems (n = 131)**	
Without detailed documentation	22 (16.8%)
With detailed documentation	109 (83.2%)
**Consequences of technical problems n = 131**	
No consequences	25 (19.1%)
Teleconsultation prolonged	105 (80.2%)
Teleconsultation terminated	1 (0.8%)
**Consequences of terminated teleconsultations n = 1**	
Rescheduled teleconsultation performed	0 (0.0%)
Rescheduled teleconsultation terminated	0 (0.0%)
**Site affected by technical problems n = 109**	
GP practice	65 (59.6%)
Neurology specialist	67 (61.5%)
**Nature of technical problems n = 109**	
Video	60 (55.0%)
Audio	85 (78.0%)

Multiple choice allowed

Details documented after adaptation of Tenedoc database

#### Process metrics and costs

According to the UMG log, the average duration of a preparatory visit including device training was 45 min. Per the Tenedoc database, the average time between a request and the start of the teleconsultation was 26.3 min, and teleconsultations lasted 26.6 min on average. The costs for each teleconsultation were between €80 and €90, without and with depreciation of the one-time expenses [21]. These costs of the NeTKoH teleconsultations were slightly above or in line with the current remuneration for teleconsultations in outpatient and inpatient settings, respectively, in the German healthcare system.

### Maintenance

The willingness of patients and GPs to continue using teleconsultations was used as an indicator for the dimension *Maintenance*. In the intervention, 469 (91.2%) out of 514 patients asserted that they would use teleconsultations again (defined as responses “strongly agree” or “agree”). This proportion of teleconsultation re-use intention was higher among male than female patients (94.1% vs. 89.3%), and above 85% across all age groups (see Appendix Table A6).

At the end of the project, 26 of 33 GPs (78.8%) stated in the exit questionnaire that they would continue to use teleconsultations in the future (see Supplementary Materials Table S1). Due to small case numbers, for data protection and privacy reasons, it was not possible to report on the characteristics of GPs who continued teleconsultations after the end of the trial.

#### Adaptations

Three months after the trial ended, 14 of the highest recruiting NeTKoH practices wanted to continue the intervention in a modified form offered by UMG two times a week on Tuesdays and Thursdays, 8 a.m. – noon. On the licensed internet platform arztkonsultation.de for teleconsultations, a link is sent from the UMG for a video call when requested by a GP. The UMG is currently remunerated for these teleconsultations via a specific teleconsultation payment scheme, while the participating GPs receive the usual remuneration as part of their GP services. Three months later, five practices were actively participating. As this adaptation (two mornings per week with some lead time) differed substantially from the original NeTKoH program (five days a week on the same day) and did not include any additional payments for GPs, this information was not considered in the *Maintenance* dimension.

### RE-AIM score

**Fig. 2:**
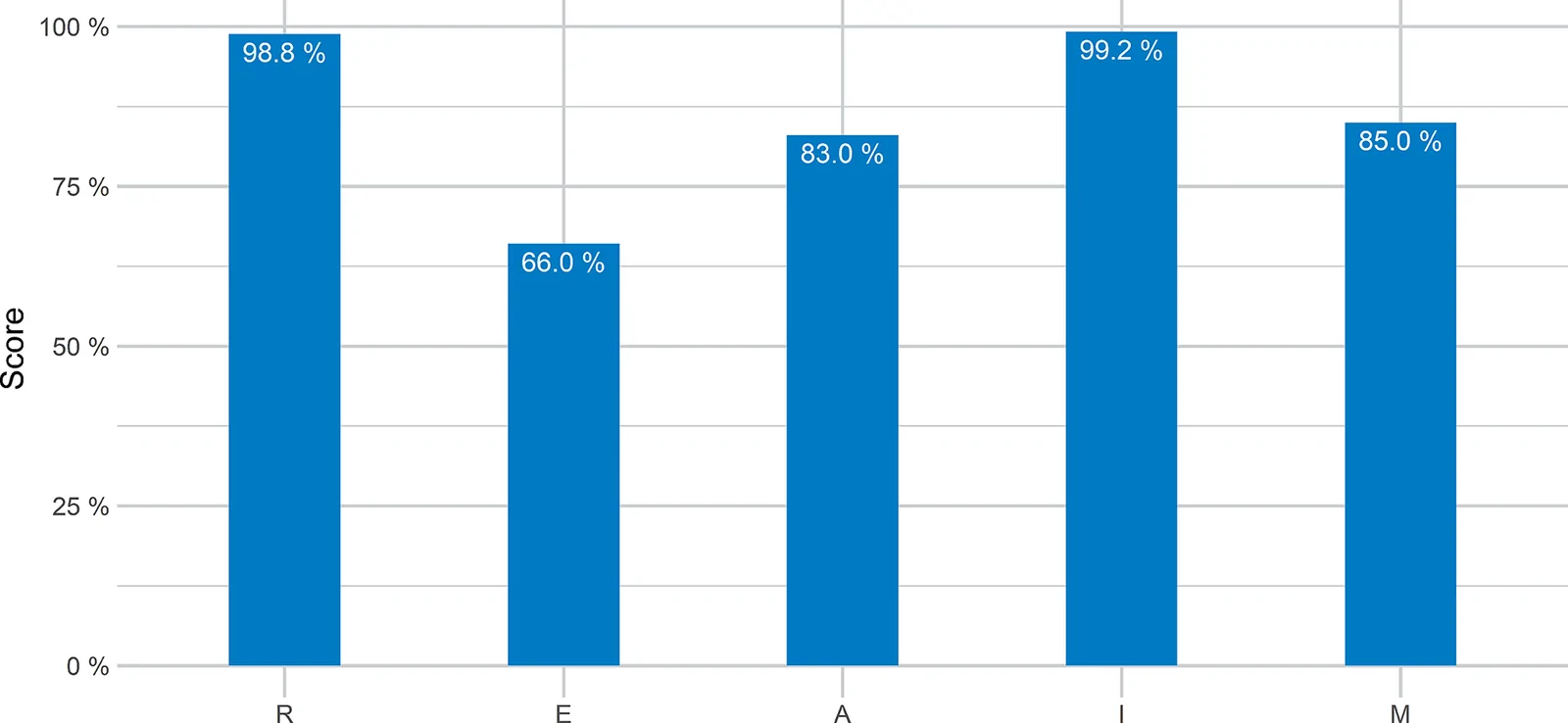
RE-AIM – Reach (**R**), Effectiveness (**E**), Adoption (**A**), Implementation (**I**), and Maintenance (**M**)

Figure 2 shows a summary of the results for each RE-AIM dimension. Aligned with RE-AIM guidance [42, 43], we used a 5-Point Scale to benchmark and interpret the scores received for each dimension: Poor < 20% – Major concerns; significant barriers or challenges are present, Fair 20–39% – Some successes noted, but substantial barriers exist that hinder progress, Moderate 40–60% – Achievements are evident, but there is room for improvement, Good 61–80% – Generally positive outcomes with minor barriers; the program is functioning well, Excellent > 80% – Exemplary performance. 

NeTKoH demonstrated “exemplary performance” (> 80%) for the RE-AIM dimensions *Reach* (98.8%), *Adoption* (83.0%), *Implementation* (99.2%), and *Maintenance* (85.0%), while the *Effectiveness* dimension was “generally positive” at 66.0% (see Fig. 2). Consistently high values for the individual metrics were found in Reach, Adoption, Implementation, and Maintenance, but Effectiveness showed mixed individual results (see Table 3): “Just-in-Time Care”, “Satisfaction/Acceptance and Recommendation” were high (98.6% and 80.9%), while “Effect Size” and “QoL” were lower (38.3% and 46.3%).

**Table 3:** RE-AIM metrics

Dimension	Item	Num.	Den.	Score
**RE-AIM Total Score**				**86.4%**
**Reach**				**9** **8.8%**
Patients reached	Patients in intervention/Patients approached in intervention	516	527	97.9%
Patients with intervention	Patients in intervention with teleconsultation/Patients in intervention	514	516	99.6%
**Effectiveness**				**66.0%**
Effect size Primary endpoint	Patients triaged to GP care (questions resolved)/Patients in intervention with teleconsultation	197	514	38.3%
Improved Quality of Life	Patients in intervention with higher QoL-index at follow-up/ Patients in intervention with teleconsultation	238	514	46.3%
Just-in-Time Care	Patients in intervention with same-day teleconsultation/ Patients in intervention with teleconsultation	507	514	98.6%
Satisfaction, acceptance, recommendation	Patients in intervention with 3 positive answers/ Patients in intervention with teleconsultation	416	514	80.9%
**Adoption**				**83.0%**
Participation	GPs participating/ GPs with an expressed interest	41	48	85.4%
Participation until the end	GPs participating until the study end/ GPs participating	33	41	80.5%
**Implementation**				**99.2%**
Equipment compliance	Teleconsultation devices installed/ Teleconsultation devices planned	34	34	100.0%
Transition compliance	Preparatory visits with device trainings performed/ Preparatory visits with device trainings planned	34	34	100.0%
Initial use:	GPs with teleconsultation(s) performed/ GPs participating until the end	32	33	97.0%
Total use	Teleconsultations performed/ Teleconsultations desired	514	516	99.6%
**Maintenance**				**85.0%**
Reuse intention (Patients)	Patients intending to use again/ Patients in intervention with teleconsultation	469	514	91.2%
Reuse intention (GPs)	GPs intending to use again/ GPs participating until the end	26	33	78.8%

## Discussion

This study explored the implementation of neurologic teleconsultations under real-world conditions in primary care in rural areas of Northeast Germany. Guided by the RE-AIM framework, we found that the NeTKoH teleconsultations achieved “excellent performance” (> 80%) for the dimensions *Reach*,* Adoption*,* Implementation*, and *Maintenance*, exceeding respective metrics found in other teleneurologic studies [44], while the *Effectiveness* dimension was “generally positive” (> 60%), albeit with mixed individual results.

Near-complete *Reach* was achieved with 98.8% of approached patients participating, most often suffering from headaches, typically in their fifties (average age 53.4 years), with a female majority (60.1%), as previously seen in similar studies [45]. However, the relatively high proportion of female patients and the comparatively young mean age may have influenced the spectrum of neurologic conditions observed and patient-reported outcomes, including QoL and should be considered when generalizing the findings to other neurologic populations. Moreover, recruitment through participating GP practices may have introduced selection bias and limited representativeness. *Adoption* by GPs was substantial at 83.0%, although only 32 out of 33 GPs completed teleconsultations with a teleneurologist of the associated university hospital, for a total of 514 analyzed interventions over 31 months. However, the total number of GPs approached or invited to participate was not documented during the pre-project setup phase, limiting the ability to assess *Adoption* and representativeness, while differential retention may have introduced participation bias. The provision of a € 100 incentive for each enrolled patient may have played a role in the observed higher adoption rate. PCPs who participated until the end of the study were mostly male (63.6%), in the age group below 45 (42.4%), and had no prior telemedicine experience. More than half of the female NeTKoH PCPs discontinued their participation, suggesting that measures should be put in place for future studies in primary care to improve participation and retention of female practitioners. As the first years of NeTKoH coincided with the COVID-19 pandemic, social distancing requirements may have contributed to the high levels of engagement among patients and PCPs, as reported in other studies [45]. However, the observation that teleconsultations in the intervention were only performed by 32 out of 33 PCPs warrants further investigation of root causes and countermeasures, including the integration into practice workflow.

Technical problems that occurred during the teleconsultations appeared manageable: Only two of the desired video conferences could not be requested; the rest were completed, predominantly on the same day (98.6%), despite technical problems in a quarter of the cases (25.5%). Satisfaction, acceptance, and recommendations for future use were very high among patients (80.9%). These findings on patient satisfaction, duration of teleconsultations, and technical issues are consistent with other studies [44–47].

Despite strong and positive implementation outcomes, short-term Effectiveness outcomes were more modest. Effectiveness for the primary endpoint was only “fair”, with 38.3% of intervention patients triaged to be managed by their GP compared to 50.7% in the control phase. However, remaining under GP care should be interpreted in the context of appropriate triage and management, as referral to an outpatient neurologist, another specialist, or hospital care when clinically indicated also represents an appropriate result. Accordingly, the observed referrals during the intervention phase, with fewer referrals to outpatient neurologists but more referrals to other specialists and hospital care compared to the control phase may indicate that teleconsultations supported more differentiated triage for complex neurologic symptoms rather than simply reducing referrals. Quality of life improvements were only “moderate”, with 46.3% among intervention patients at follow-up. However, the trial endpoint analysis showed no significant difference in QoL changes between intervention and control [19]. Given the complexity of neurologic conditions and the short three-month follow-up period, substantial QoL changes were not to be expected after a single teleconsultation, but QoL was a patient-oriented secondary endpoint defined in the study protocol. To better assess and understand the benefits of neurologic teleconsultations in primary care, future studies should also include resources for an independent neurologic expert review of the appropriateness of the triage and referrals, as in other studies [46]. In addition, prolonged or repeated follow-ups are needed to assess sustained changes in QoL and the course of the underlying neurologic complaints. The higher proportion of hospital referrals may also reflect a more cautious approach by teleneurologists when assessing patients remotely [19].

Costs of each teleconsultation were within the range of current payment schemes for teleconsultations (including patient assessments) in the German healthcare system. Although high intention-based *Maintenance* levels suggested an excellent outlook for sustainability of NeTKoH, long-term sustainability remains to be established. Future projects should consider tailoring the temporal availability of teleconsultations to meet GP needs.

### Strengths and limitations

As a strength of our study under real-world-conditions, mindful of workflow constraints and based on UMG’s prior experience with teleneurologic and primary care projects, we used paper questionnaires for patients and GPs, derived from an extensive literature search and piloted with the stakeholders, and we included the EQ-5D-5​L as a validated instrument. For practical reasons, the number of patients approached was recorded on self-completed paper lists by the GPs. The effectiveness analysis followed the ITT principle and the predefined plan, and an analysis of the actual follow-up (per protocol) was not intended as part of this implementation study. As a limitation, identification and recruitment bias cannot be ruled out, as GPs knew about the study phase before approaching patients for possible enrollment. Data were rigorously assessed for quality and completeness through numerous plausibility and validation checks, and corrections were provided or confirmed by the principal investigator at UMG, a research institution that is very experienced with clinical data management and curation, including study logs, databases, and forms. Furthermore, we were able to report on all five RE-AIM dimensions, which is not always possible [42]. *Maintenance* was limited to intentions to continue rather than actual post-study utilization data, as longer-term assessment was not feasible within the funding and project timeframe, following RE-AIM guidance [48]. Although the adapted continuation program differed substantially from NeTKoH, the decrease from 14 to 5 participating practices suggests that long-term *Maintenance* may be more challenging.

No special consideration was given to low resource populations, and access depended on practice participation and on patient characteristics, such as acceptance. Older or disadvantaged patients might therefore have been underrepresented. However, enrollment in GP practices was intended to maximize representativeness of the local primary care population, while the practice-based setup avoided digital inequality by not requiring patients to have their own devices or technical skills. Future research should strive to further improve inclusiveness and equity, including in surveys and questionnaires. Although we aimed for inclusive reporting, we were subject to data protection regulations and therefore could not report on characteristics on some subgroups, nor on patients that did not give consent to participate, and we could only compare the study population to regional and national statistics. Additionally, a comparison between the NeTKoH population and the patient population in NeTKoH practices, in terms of representativeness, was not feasible due to lack of resources.

We also lacked information on aspects of the GP recruitment process for *Adoption*, which we mitigated by comparing characteristics to regional and national official data. While the participating GPs were representative in terms of practice form and size, they differed in terms of age and sex from the GPs in the state and in Germany. As a limitation, it must be assumed that they further differ from the non-participating GPs, e.g., regarding spatial or personnel resources, prevalence of neurologic diseases in their practices, interest and connection to population health research, so that self-selection bias cannot be ruled out. The characteristics of GPs that discontinued their participation and of GPs with “willingness to continue” as an indicator for *Maintenance* (setting level) could not be reported due to low case numbers and privacy concerns. However, information on the characteristics of patients who would continue to use the intervention is available at the individual metric level for *Maintenance*.

Furthermore, no data could be collected on the satisfaction or acceptance of the medical assistants or the teleneurologists for the item *Adoption* (staff level), in view of concerns regarding disruption of practice workflow. Since medical assistants and teleneurologists are active users of the intervention, future projects should broadly include the perspectives of all users and stakeholders of the intervention during planning, implementation, and evaluation.

Also, due to the structure of the funding framework and time constraints, we were unable to use mixed-methods approaches, such as qualitative interviews, for a more comprehensive assessment of barriers and facilitators. While the structured questionnaires captured patient satisfaction and acceptability, they did not allow for an in-depth exploration of patient experiences. Qualitative approaches could have contributed to a better understanding of recruitment and provided further insights into the population reached by the intervention (*Reach*), patient experiences, satisfaction, and *Adoption* at the setting level. However, corresponding qualitative work is ongoing outside of the project by the UMG as part of a doctoral thesis.

### Lessons learned

Overall, several lessons for future implementation and scale-up can be drawn from NeTKoH. During the pre-implementation phase, organization, provider, and staff recruitment should be systematically documented, e.g., the number of GPs approached and invited to participate in primary care studies. Patient and GP recruitment and retention should be considered early, with particular attention to female practitioners. Although the reasons for the observed differences in retention by GP sex could not be determined from our data, potential strategies could include minimizing additional practice workload and providing flexible working and implementation arrangements [49]. Stakeholder engagement throughout planning, implementation, and evaluation should include patients, GPs, medical assistants as well as teleneurologists and consider inclusiveness, workflows, workload, remuneration schemes, and practitioner needs. For medical assistants and teleneurologists, standardized onboarding and training, clear workflows and responsibilities, as well as readily available technical guidance and support may facilitate implementation. The perspectives of medical assistants and teleneurologists should also be systematically assessed to identify barriers related to workflow, workload, usability, and training needs. Routine technical checks, standardized troubleshooting procedures, and systematic monitoring of technical problems may reduce recurring technical failures and further improve implementation reliability [50]. Future evaluations should carefully select *Effectiveness* outcomes and include independent neurologic expert assessment of patient conditions and the appropriateness of triage decisions. At the structural level, longer funding periods would facilitate multiple or longer follow-ups to assess sustained *Effectiveness* and actual post-study *Maintenance*. Greater availability of public secondary data via data infrastructures on relevant patient and GP populations would also facilitate assessment of *Reach*,* Adoption*, and representativeness. Where such data is unavailable, collecting appropriate reference data in the settings would require additional resources. Although transferability to other specialties was beyond the scope of NeTKoH, the general model of real-time specialist support integrated into primary care may also be relevant to other specialties, subject to specialty-specific adaptation and evaluation.

## Conclusions

The implementation of teleconsultations in NeTKoH achieved excellent implementation outcomes for the *Reach*, *Adoption*, *Implementation*, and *Maintenance* dimensions of the *RE-AIM* approach, while values for *Effectiveness* were more modest. The results of the evaluation suggest that it is feasible to integrate neurologic teleconsultations into routine primary care and to provide timely access to neurologic specialist services for patients in rural and underserved communities. While the proportion of patients managed solely by GPs did not increase in the short term, teleconsultations supported timely specialist assessment and triage, including referrals to further care where indicated. Although the representativeness of the NeTKoH patients and GPs could only be partially assessed, the patient population was similar to the regional population, and the GPs were comparable in many respects to GPs in the state of Mecklenburg-Western Pomerania.

In summary, the RE-AIM framework provided relevant insights into the implementation of neurologic teleconsultations in primary care in underserved areas, which justifies a roll-out in Mecklenburg-Western Pomerania and can guide a dissemination trial across other states in Germany for a future scale-up.

## Supplementary Information

Below is the link to the electronic supplementary material.

Supplementary Material 1

## Data Availability

The analysis scripts are available from the authors at the Institute of Public Health upon reasonable request. The data gathered and compiled in this study is not publicly available and cannot be shared due to German data protection laws and the Social Code Book (SGB). In accordance with the patient information from the approved informed consent material, study data must remain confidential within the project consortium and cannot be shared with third parties.

## Footnotes

1 Per the approved NeTKoH data protection protocol, and compliant with data privacy regulations, we did not report groups < 5.

## Declarations

### Standards of reporting

This study reporting adheres to the RE-AIM framework [14] and follows the Standards for Reporting Implementation Studies (StaRi) [23] and the expanded RE-AIM CONSORT figure [24].

## Abbreviations

CONSORT: Consolidated standards of reporting trials
GP: General practitioner
LTFU: Lost to follow-up
NeTKoH: Neurologic teleconsultation with general practitioners to strengthen specialist care in Western Pomerania
QoL: Quality of life
RE-AIM: Reach-Effectiveness-Adoption-Implementation-Maintenance
SD: Standard deviation
StaRi: Standards for reporting implementation studies
UMG: University Medical Center Greifswald
vs: versus
WONCA: World organization of national colleges academies and academic associations of general practitioners/family physicians
