# Supplementary material for: The implementation of neurologic teleconsultations in rural primary care in Northeastern Germany (NeTKoH): an evaluation guided by the RE-AIM framework
Source: BMC Health Serv Res. 2026 Nov 10;26:1562. doi: 10.1186/s12913-026-16062-0 (PMC13757052; doi:10.1186/s12913-026-16062-0)
Supplement: Supplementary file 1 [file 12913_2026_16062_MOESM1_ESM.docx]

# Corresponding author: [kerstin.wainwright@charite.de](mailto:kerstin.wainwright@charite.de)

**Appendix**

**Table A1:** RE-AIM-metrics and data sources

| **RE-AIM Dimension** | **Data Source** |
| --- | --- |
| **Reach**  Does the intervention reach the target population? | |
| % Patients willing to participate: Number of patients in intervention/Number of patients approached in intervention | GP screening logs |
| % Patients actively participating: Number of Patients in intervention with teleconsultation/Number of patients in intervention | Tenedoc database |
| Characteristics/representativeness of patients reached, and with follow-up vs. lost to follow-up | Patient questionnaire, GP questionnaire, Tenedoc database |
| Reasons for not receiving the teleconsultation (patients) | Tenedoc database, UMG log |
| **Effectiveness**  Does the intervention achieve the intended goals/outcomes? | |
| % Effect size: Number of patients triaged to GP care (questions resolved)/Number of Patients in intervention with teleconsultation | Tenedoc database |
| % Improved Quality of Life: Number of patients in intervention with higher QoL-index at follow-up (EQ-5D-5L)/Number of patients in intervention with teleconsultation | Patient questionnaire, paper-based and in phone interview |
| % Just-in-time care: Number of patients in intervention with same-day teleconsultation/Number of patients in intervention with teleconsultation | Tenedoc database |
| % Satisfaction, acceptance, recommendation: Number of patients in intervention with 3 positive answers/Number of patients in intervention with teleconsultation | Patient questionnaire |
| **Adoption**  To what extent do the service providers/settings involved participate in the intervention? | |
| % Actual participation: Number of GPs participating/Number of GPs with an expressed interest | UMG log |
| % Participation until the end: Number of GPs participating until the study end/Number of GPs participating | UMG log |
| Characteristics/representativeness of GPs who participated, and who participated until the end vs. discontinued participation | Entry questionnaire |
| Reasons for discontinued participation (GPs) | UMG log |
| **Implementation**  To what extent are the activities implemented? | |
| % Fidelity – Equipment compliance: Number of teleconsultation devices installed/Number of teleconsultation devices planned | UMG log |
| % Fidelity – Transition compliance: Number of preparatory visits with device trainings performed/Number of preparatory visits with device trainings planned | UMG log |
| % Fidelity - Initial use: Number of GPs with teleconsultation(s) performed/Number of GPs participating until the end | Tenedoc database |
| % Fidelity – Total use: Number of teleconsultations performed/Number of teleconsultations desired | Tenedoc database |
| **Maintenance**  To what extent can the intervention be sustainably integrated into routine processes? | |
| % Reuse intention (as an indicator): Number of patients intending to use again/Number of patients in intervention with teleconsultation | Patient questionnaire |
| % Reuse intention (as an indicator): Number of GPs intending to use again/Number of GPs participating until the end | Exit questionnaire |

***Table A2:*** Reach – Neurologic symptoms of intervention patients with and without loss to follow-up

| **Symptoms of Patients in the intervention** | **Followed Up (N=448)** | **Lost to Follow-Up (N=66)** | **Total (N=514)** |
| --- | --- | --- | --- |
| Headache(s) | 146 (32.6%) | 24 (36.4%) | 360 (36.6%) |
| Sensory disorder(s) | 124 (27.7%) | 15 (22.7%) | 234 (23.8%) |
| Vertigo | 118 (26.3%) | 14 (21.2%) | 229 (23.3%) |
| Other pain(s) | 44 (9.8%) | <5 | 140 (14.2%) |
| Other symptom(s) | 43 (9.6%) | <5 | 115 (11.7%) |
| Motor deficit(s) | 35 (7.8%) | <5 | 104 (10.6%) |
| Vision impairment(s) | 35 (7.8%) | 6 (9.1%) | 86 (8.7%) |
| Movement disorder(s) | 29 (6.5%) | 8 (12.1%) | 80 (8.1%) |
| Language, speech, or swallowing disorder(s) | 10 (2.2%) | <5 | 32 (3.3%) |
| Consciousness clouding or disorders(s) | 8 (1.8%) | <5 | 27 (2.7%) |
| Character changes | 6 (1.3%) | <5 | 25 (2.5%) |
| Multiple choices allowed | |  | |

**Table A3:** Recommendations regarding further care (triage)

| **Patients in the intervention** | **Total (N=514)** |
| --- | --- |
| **Triage** |  |
| Primary Care Physician | 197 (38.3%) |
| Neurologist | 187 (36.4%) |
| Other specialist physician | 61 (11.9%) |
| Hospitalization | 66 (12.8%) |
| Other(s) | <5 |

**Table A4:** Patient satisfaction with, acceptance of, and recommendation of teleconsultations

| **Patients in the intervention (N=514)** | **Strongly disagree** | **Disagree** | **Neutral** | **Agree** | **Strongly agree** | **Missing** |
| --- | --- | --- | --- | --- | --- | --- |
| I am satisfied overall with the quality of health care provided via teleconsultations | 1 (0.2%) | 6 (1.2%) | 51 (9.9%) | 154 (30.0%) | 301 (58.6%) | 1 (0.2%) |
| Teleconsultations are acceptable for my health care | 2 (0.4%) | 13 (2.5%) | 52 (10.1%) | 161 (31.3%) | 285 (55.4%) | 1 (0.2%) |
| I will recommend teleconsultations | 4 (0.8%) | 5 (1.0%) | 47 (9.1%) | 131 (25.5%) | 325 (63.2%) | 2 (0.4%) |

**Table A5:** Characteristics of the primary care physicians at the start of their participation

| **Primary care physicians who participated** **until the end of the NeTKoH study phase** | **Total (N=33)** |
| --- | --- |
| **Sex** |  |
| Female | 12 (36.4%) |
| Male | 21 (63.6%) |
| **Age [years]** |  |
| Mean (SD) | 48.2 (9.1) |
| Median [Min; Max] | 47.0 [34; 65] |
| **Age group** |  |
| < 45 years | 14 (42.4%) |
| 45-54 years | 12 (36.4%) |
| ≥ 55 years | 7 (21.2%) |
| **Years of practice** |  |
| Mean (SD) | 19.5 (9.0) |
| Median [Q1; Q3] | 17.0 [13; 25] |
| **Years of practice in Mecklenburg-Western Pomerania** |  |
| Mean (SD) | 9.9 (7.7) |
| Median [Q1; Q3] | 8.0 [5; 13] |
| **Telemedical experience** |  |
| Yes | 10 (30.3%) |
| No | 23 (69.7%) |
| **Type of practice** |  |
| Individual | 21 (63.6%) |
| Other | 12 (36.4%) |
| **Number of cases per quarter** |  |
| < 750 | <5 |
| 750-999 | 8 (24.2%) |
| 1000-1500 | 18 (54.5%) |
| > 1500 | <5 |

**Table A6:** Maintenance – Willingness to reuse teleconsultations, differentiated by demographics

| **Patients in the intervention** | **N** | **Willing to use again** |
| --- | --- | --- |
| **Total** | 514 | 469 (91.2%) |
| **Sex** |  |  |
| Female | 309 | 276 (89.3%) |
| Male | 205 | 193 (94.1%) |
| **Age group** |  |  |
| 18-29 years | 80 | 74 (92.5%) |
| 30-49 years | 133 | 126 (94.7%) |
| 50-69 years | 172 | 151 (87.8%) |
| 70-79 years | 77 | 71 (92.2%) |
| ≥ 80 years | 52 | 47 (90.4%) |

# Supplementary Materials

**Table S1:** English translations of the questions related to (satisfaction with) the teleconsultations, from the patient and GP questionnaires

| **Patients, immediately after their teleconsultation** | **Answer options** |
| --- | --- |
| **Technical aspects** | Strongly disagree, Disagree, Neutral, Agree, Strongly agree |
| I was able to easily talk to the tele-neurologist. |  |
| I was able to clearly hear the tele-neurologist. |  |
| I was able to see the tele-neurologist as if we met in person. |  |
| The tele-neurologist was able to examine me by video. |  |
| **Comfort and knowledge** |  |
| The tele-neurologist understood my health condition. |  |
| I felt comfortable during the tele-neurologist examination. |  |
| I know how I will continue to receive medical care. |  |
| I know what to do if my health condition deteriorates. |  |
| **Perceived benefits** |  |
| Teleconsultations improve my access to healthcare. |  |
| Teleconsultations help me save time. |  |
| Teleconsultations help me save trips. |  |
| Teleconsultations meet my needs. |  |
| **Satisfaction and future use** |  |
| Teleconsultations are acceptable for my health care. |  |
| I would use teleconsultations again. |  |
| I will recommend teleconsultations. |  |
| I am satisfied overall with the quality of health care provided via teleconsultations. |  |
| **GPs, immediately after each teleconsultation** | **Answer options** |
| **Reliability and quality** | Strongly disagree, Disagree, Neutral, Agree, Strongly agree, Not applicable |
| The video quality was flawless. |  |
| The audio quality was flawless. |  |
| Any technical problems were quickly resolved. |  |
| **Ease of use** |  |
| The teleconsultation device was easy to use. |  |
| The teleconsultation was easy to integrate into my doctor-patient relationship. |  |
| The teleconsultation was easy to integrate into my practice workflow. |  |
| **Satisfaction and future use** |  |
| The teleconsultation was an acceptable way to care for this patient. |  |
| I was satisfied with today’s teleconsultation. |  |
| The teleconsultation was helpful for this patient. |  |
| **GPs, exit questionnaire at the end of the study** | **Answer options** |
| I was satisfied overall with the teleconsultations. | Strongly disagree, Disagree, Neutral, Agree, Strongly agree |
| I will recommend teleconsultations in primary care practices. |  |
| I would use teleconsultations again. |  |
| In general, the recommendations were helpful in solving the patient’s neurological problems. |  |
| Teleconsultations are acceptable in providing care to patients. |  |
| Teleconsultations in primary care practices deliver an added patient value. |  |
